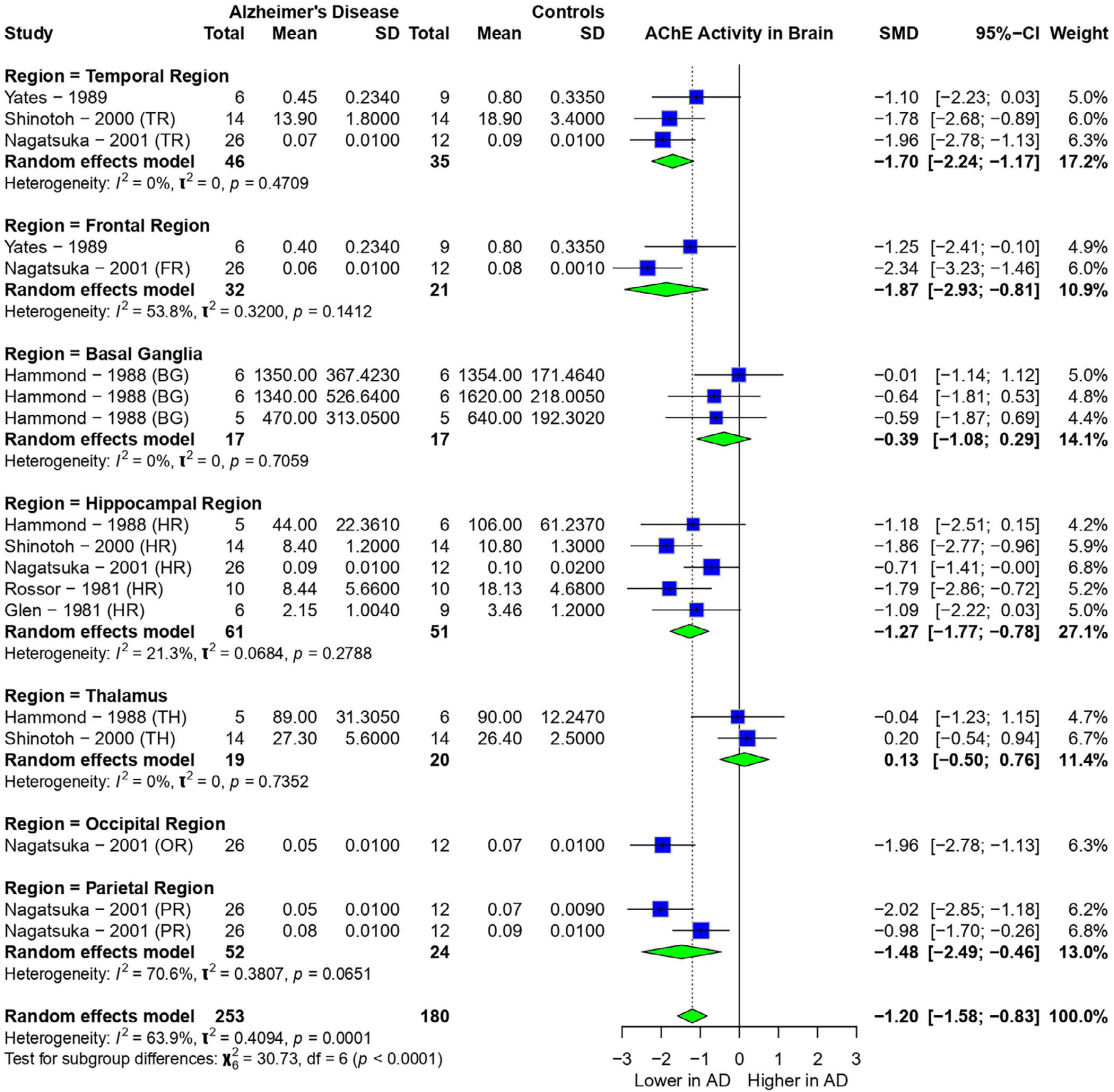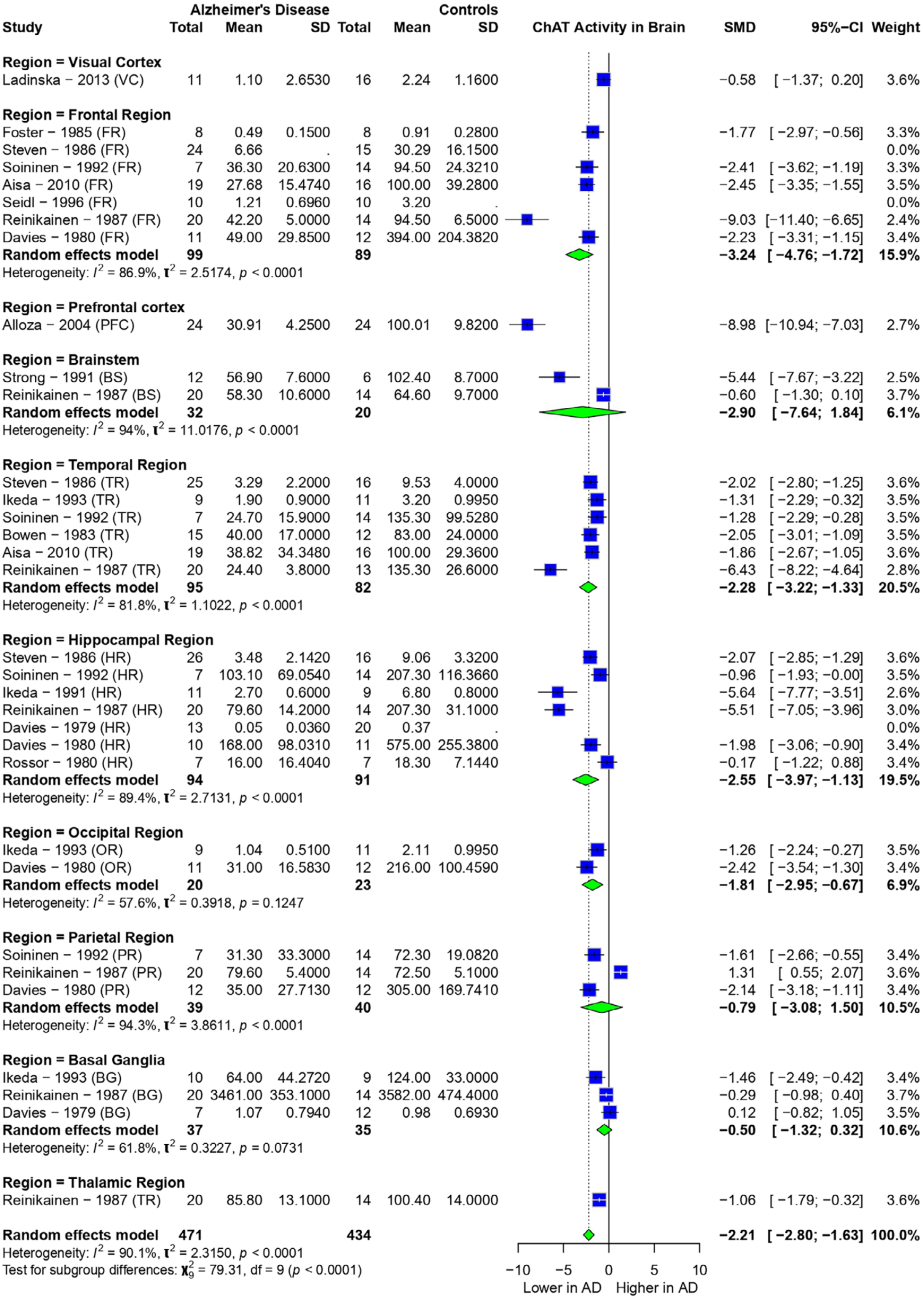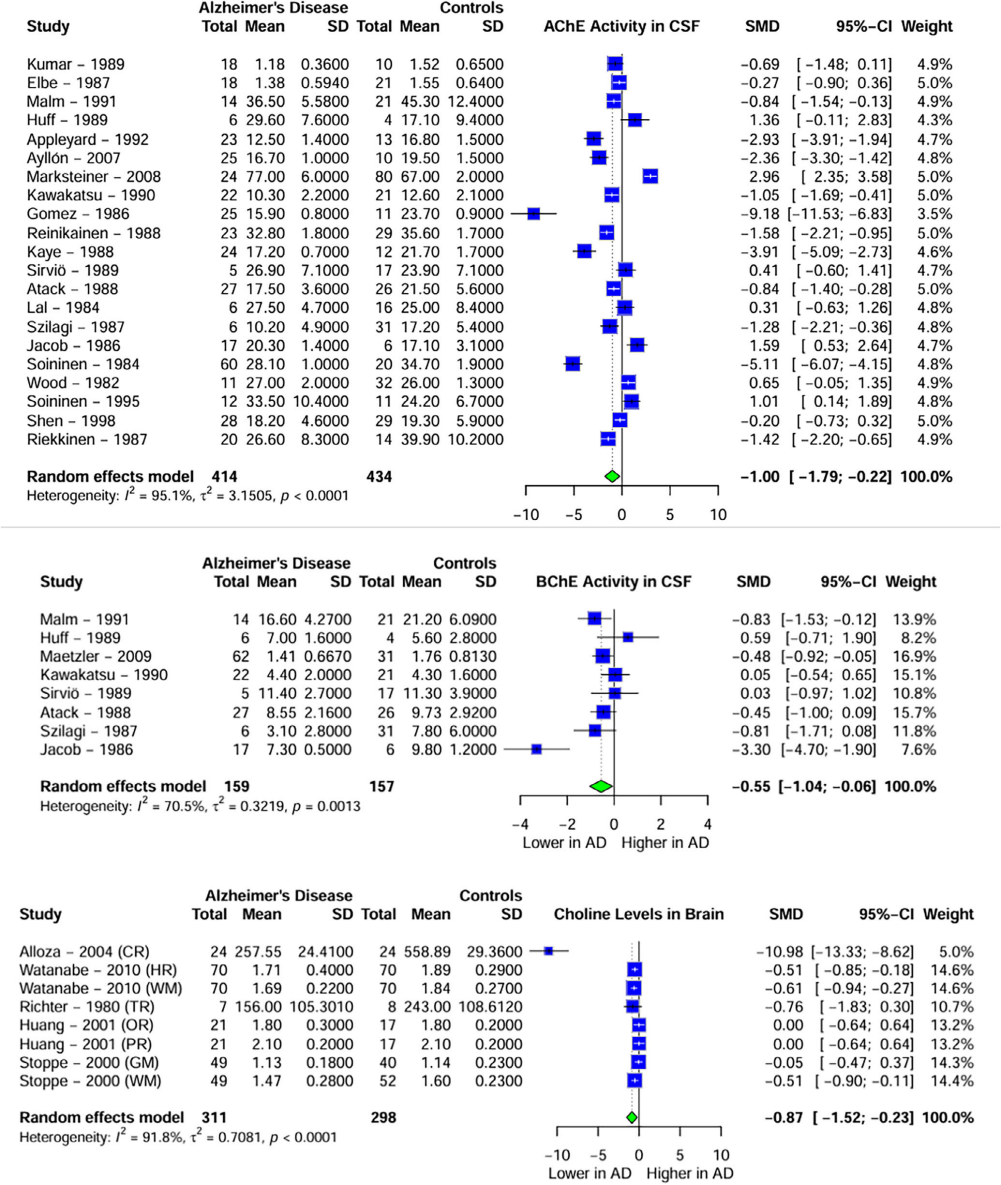# Cholinergic Dysregulation in Alzheimer’s Disease: A Meta‐Analysis of Enzymatic Activity and Regional Alterations in Brain and Cerebrospinal Fluid

**DOI:** 10.1002/alz70862_109919

**Published:** 2025-12-23

**Authors:** Muneeb Ahmad Muneer, Harshita Agarwal, Poorvikha Gowda, Mohammad Orooj Azmi, Parshant Yadav, Victor Ghosh, Mohammad Hassan, Anmol Kaur, Vinay Suresh, Ahmed Y. Azzam, Mainak Bardhan

**Affiliations:** ^1^ Allama Iqbal Medical College, Lahore, Punjab Pakistan; ^2^ Institute of Post Graduate Medical Education and Research, Kolkata, West Bengal India; ^3^ St John’s Medical College, Bangalore, Karnataka India; ^4^ Maulana Azad Medical College, New Delhi, Delhi India; ^5^ Andhra Medical College, Visakhapatnam, Andhra Pradesh India; ^6^ Katihar Medical College, Katihar, Bihar India; ^7^ Lady Hardinge Medical College, New Delhi, Delhi India; ^8^ King George's Medical University, Lucknow, Uttar Pradesh India; ^9^ Montefiore‐Einstein Cerebrovascular Research Lab, Albert Einstein College of Medicine, New York, NY USA; ^10^ Miami Cancer Institute, Baptist Health South Florida, USA, Miami, FL USA

## Abstract

**Background:**

Alzheimer's disease (AD), the leading cause of dementia, is characterized by β‐amyloid plaques, tau tangles, and early cholinergic dysfunction. This meta analysis examines changes in Butyrylcholinesterase (BChE), Acetylcholinesterase (AChE), Choline Acetyltransferase (ChAT), and choline levels in brain regions and cerebrospinal fluid (CSF) of AD patients versus healthy controls, highlighting their association with cognitive impairment.

**Method:**

We searched MEDLINE, EMBASE, Cochrane, and Scopus for studies on BChE, AChE, ChAT activity, and choline levels in CSF and brain regions of AD patients, following PRISMA guidelines. Meta‐analysis used R's 'meta' package with inverse variance weighting to calculate mean concentrations and Standardized Mean Differences (SMDs). Heterogeneity was assessed using I² and τ², with τ² estimated via restricted maximum‐likelihood and Q‐profile methods.

**Result:**

AChE showed significant SMD of ‐1.00 (95% CI: ‐1.79 to ‐0.22, I² = 95.1%) in 22 studies (AD 414, 434 controls) in CSF. For brain regions, AChE showed significant SMD of ‐1.20 (95% CI: ‐1.58 to ‐0.83, I² = 63.9%) in 6 studies (AD 253, 180 controls), with the most significant SMDs of ‐1.70 (temporal), ‐1.87 (frontal), ‐1.48 (parietal), and ‐1.27 (hippocampal). BChE showed significant SMD of ‐0.55 (95% CI: ‐1.04 to ‐0.06, I² = 70.5%) in 8 studies (AD 159, 157 controls) in CSF. ChAT showed significant SMD of ‐2.21 (95% CI: ‐2.80 to ‐1.63, I² = 90.1%) in 13 studies (AD 471, 434 controls), with the most significant SMDs of ‐3.24 (frontal), ‐2.55 (hippocampal), ‐2.28 (temporal), and ‐1.81 (occipital). Choline showed a significant SMD of ‐0.87 (95% CI: ‐1.52 to ‐0.23, I² = 91.8%) in 5 studies (AD 311, 298 controls).

**Conclusion:**

AChE showed significantly reduced activity in CSF and particularly in the temporal, frontal, hippocampal, and parietal brain regions. BChE exhibited significant reduction in CSF activity, and choline levels were significantly decreased in brain regions. ChAT showed significant reduction in the frontal, temporal, hippocampal, and occipital brain regions. Further research is required to validate these findings.